# Effect of interlayer interactions on exciton luminescence in atomic-layered MoS_2_ crystals

**DOI:** 10.1038/srep29813

**Published:** 2016-07-15

**Authors:** Jung Gon Kim, Won Seok Yun, Sunghwan Jo, JaeDong Lee, Chang-Hee Cho

**Affiliations:** 1 Department of Emerging Materials Science, DGIST, Daegu 42988, South Korea

## Abstract

The atomic-layered semiconducting materials of transition metal dichalcogenides are considered effective light sources with both potential applications in thin and flexible optoelectronics and novel functionalities. In spite of the great interest in optoelectronic properties of two-dimensional transition metal dichalcogenides, the excitonic properties still need to be addressed, specifically in terms of the interlayer interactions. Here, we report the distinct behavior of the A and B excitons in the presence of interlayer interactions of layered MoS_2_ crystals. Micro-photoluminescence spectroscopic studies reveal that on the interlayer interactions in double layer MoS_2_ crystals, the emission quantum yield of the A exciton is drastically changed, whereas that of the B exciton remains nearly constant for both single and double layer MoS_2_ crystals. First-principles density functional theory calculations confirm that a significant charge redistribution occurs in the double layer MoS_2_ due to the interlayer interactions producing a local electric field at the interfacial region. Analogous to the quantum-confined Stark effect, we suggest that the distinct behavior of the A and B excitons can be explained by a simplified band-bending model.

Over the last several years, two-dimensional atomic-layered transition metal dichalcogenides (TMDCs) such as molybdenum disulphide (MoS_2_) and tungsten disulphide (WS_2_) have been intensively studied due to their fascinating electronic and optical properties. In particular, TMDCs exhibit intriguing optical properties due to their two-dimensionality, including strong exciton luminescence^1^,^2^,^3^, large exciton binding energy^4^, and valley exciton selection rules^5^,^6^. In atomic-layered MoS_2_, two direct optical transitions of the A and B excitons are observed at ~1.8 eV (A exciton) and ~2 eV (B exciton) and are attributed to the valence band splitting due to the spin-orbit-coupling (SOC) effect^7^. It has been reported that the luminescence quantum yield of monolayer MoS_2_ is strongly enhanced by the emergence of direct bandgap and the quantum confinement effect perpendicular to the layer plane^2^,^3^,^8^. Although the exciton-related phenomena in two-dimensional TMDC systems are of great interest, the intrinsic properties of excitons in TMDCs are basically unexplored. The interlayer interaction is one of the key parameters for determining the excitonic properties of layered TMDCs because TMDCs naturally form a homo- or hetero-structure in many practical testbeds and applications.

Previous theoretical and experimental studies have revealed that the twisted double-layer graphene system significantly alters the electronic band structure and optical properties due to the change in interlayer interactions as a function of the twisted angle^9^,^10^,^11^,^12^. Furthermore, the double-layer MoS_2_ shows largely modulated excitonic luminescence, depending on the twisted angle between the top and bottom layers^13^. The luminescence intensity and energy can be tuned with the twisted angles, and the different types of excitons, such as neutral or charged excitons, experience very different degrees of interlayer interactions at a given angle. As such, the interlayer interactions in homo- and hetero-structures play a crucial role in determining the excitonic properties of the layered TMDCs.

Here, we report the effect of interlayer interactions on exciton luminescence in atomic-layered MoS_2_ crystals. The distinct behavior of the A and B excitons in the presence of interlayer interactions was investigated by examining single (1L) and double (2L) layer MoS_2_ crystals. Micro-photoluminescence spectroscopic studies revealed that the interlayer interactions in the 2L-MoS_2_ crystals cause the emission quantum yield of the A exciton to drastically change, whereas that of the B exciton remains nearly constant for both the 1L- and 2L-MoS_2_ crystals. Based on the first-principles density functional theory (DFT) calculations, we found that a significant charge redistribution occurs in 2L-MoS_2_ due to the interlayer interactions producing a local electric field at the interfacial region. Analogous to the quantum-confined Stark effect, we suggest that the distinct behavior of the A and B excitons can be explained by a simplified band-bending model.

## Results and Discussion

**Figure 1:**
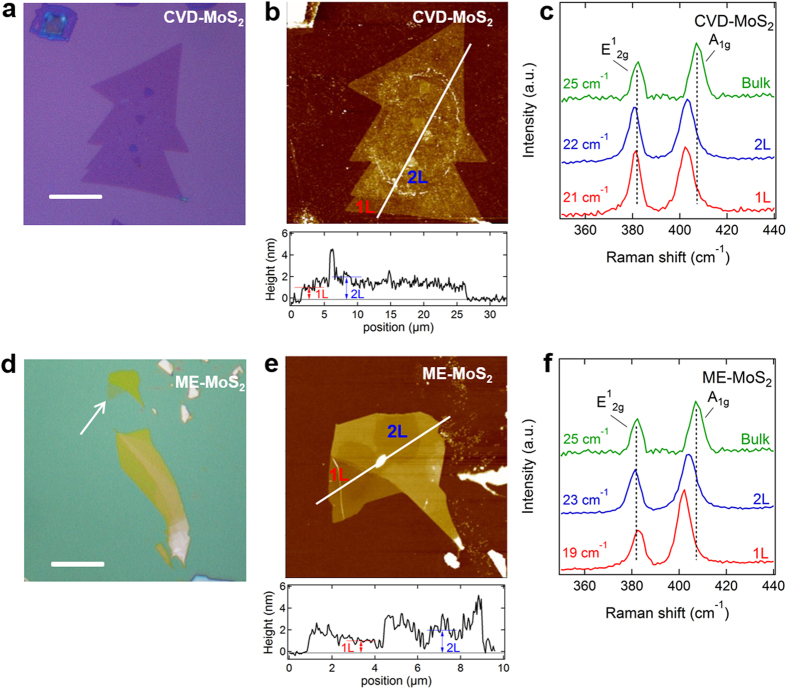
Thickness identification of atomic-layered MoS_2_ crystals. (**a**,**b**) Optical microscope (**a**) and AFM (**b**) images of the prepared CVD-MoS_2_ crystals. AFM height profile, which was recorded along the white line, is also shown in (**b**). (**c**) Raman spectra of the 1L and 2L CVD-MoS_2_ crystals. Vertical dashed lines indicate the peak positions corresponding to the in-plane (E^1^_2g_) and out-of-plane (A_1g_) phonon modes of exfoliated bulk MoS_2_ for comparison. (**d**,**e**) Optical microscope (**d**) and AFM (**e**) images of the ME-MoS_2_ crystals. Scale bars in (**a**,**d**) indicate 10 μm. (**f**) Raman spectra of the 1L and 2L ME-MoS_2_ crystals.

Figure 1 shows thin MoS_2_ crystals that were prepared by the chemical vapor deposition (hereafter CVD-MoS_2_) and mechanical exfoliation (ME-MoS_2_) methods, as shown in the upper (Fig. 1a–c) and lower (Fig. 1d–f) panels, respectively (see Methods). Typical sizes of the MoS_2_ crystals ranged from 10 to 30 μm, as confirmed by optical (Fig. 1a,d) and atomic force microscope (AFM; Fig. 1b,e) images. Single-layered MoS_2_ crystals can be visualized even in the optical microscope image because of the optical contrast between the atomic layer of the MoS_2_ crystal and the SiO_2_/Si substrate^14^. The MoS_2_ layer thickness was measured using the AFM step height profiles, which revealed that both the CVD-MoS_2_ and ME-MoS_2_ crystals have the thickness of ~1 nm and ~2 nm for the 1L and 2L regions, respectively. The layer thickness can also be characterized by Raman spectroscopy. The frequency difference between the optical phonon modes of A_1g_ (out-of-plane atomic vibration) and E^1^_2g_ (in-plane atomic vibration) is decreased when the thickness of the MoS_2_ crystal is decreased to a few atomic layers compared to that of the bulk MoS_2_ crystals (~25 cm^−1^). As shown in Fig. 1c,f the frequency difference for the 1L- and 2L-ME-MoS_2_ was measured to be 19 cm^−1^ and 23 cm^−1^, respectively, while that for the 1L- and 2L-CVD-MoS_2_ was 21 cm^−1^ and 22 cm^−1^, respectively (see Supplementary Information, Figs S1 and S2). The measured values for the frequency difference, depending on the number of layers, are in good agreement with previously reported Raman data^15^,^16^. It is known that the E^1^_2g_ phonon mode softens with an increasing number of layers but that the A_1g_ phonon mode is stiffened by the interlayer interactions, as observed in our Raman data.

**Figure 2:**
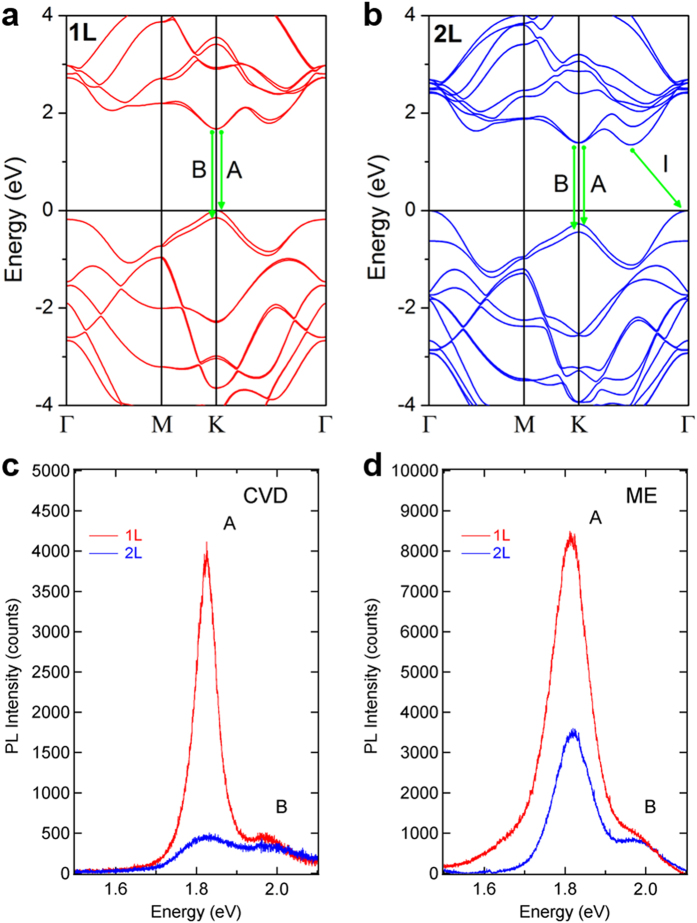
Electronic band structure and exciton luminescence spectra. (**a,b**) Electronic band structures of 1L- (**a**) and 2L-MoS_2_ (**b**) obtained by DFT calculations. Direct optical transitions from the conduction band to the split valence bands at K point are denoted by A (A exciton) and B (B exciton) along with green arrows. For 2L-MoS_2_, indirect optical transition is also indicated by I, as shown in (**b**). (**c**,**d**) Photoluminescence spectra of the CVD- (**c**) and ME-MoS_2_ (**d**) crystals measured at an excitation energy of 2.7 eV. Red and blue spectra denote 1L- and 2L-MoS_2_, respectively.

To understand optical transitions in the 1L- and 2L-MoS_2_ crystals, DFT calculations were employed to obtain electronic band structures, as shown in Fig. 2a,b. For the 1L-MoS_2_ crystals, direct optical transitions are expected between the conduction band minimum and the two valence band maxima at K point, which split due to the SOC effect^7^. The two direct optical transitions correspond to the A and B exciton states. For 2L-MoS_2_, the valence band maximum appears at Γ point, which opens indirect channels for optical transitions. Figure 2c,d exhibit the photoluminescence spectra of both the CVD- and ME-MoS_2_ crystals, as measured by micro-photoluminescence spectroscopy, which shows that the A and B exciton energies peaked at 1.82 and 1.98 eV, respectively. For both the CVD- and ME-MoS_2_ crystals, the energy difference between the A and B excitons was estimated to be 160 meV, which is in agreement with previously reported values^3^,^6^,^17^. One remarkable feature is that when the layer thickness decreases from 2L to 1L, the photoluminescence intensity of the A exciton is strongly enhanced, but that of the B exciton remains nearly constant for both the CVD- and ME-MoS_2_ crystals. Strongly enhanced photoluminescence has been reported in 1L-MoS_2_ and explained by indirect to direct bandgap crossover when decreasing the layer number from 2L to 1L. Although the strong enhancement of the A exciton in 1L-MoS_2_ can be understood by the indirect to direct bandgap crossover, the stark contrast of the B exciton showing a nearly constant intensity for both 1L- and 2L-MoS_2_ cannot be explained, which suggests that the simple scenario of indirect to direct bandgap crossover is not fully responsible for the excitonic properties of the layered MoS_2_ crystals. However, the nearly constant intensity of B exciton may be explained within the frame of indirect to direct bandgap crossover only in an exceptional case where the hole population in the valence state of B-exciton is similar for both the 1L- and 2L-MoS_2_ under optical excitation. In contrast, as recent experimental works have shown that the exciton luminescence of 1L-MoS_2_ can be significantly altered by the interaction with substrates^3^,^18^, van der Waals interactions can play a major role in determining the excitonic properties of 2-dimensional materials. The interlayer interactions observed in layered TMDCs can produce an internal electric field at the van der Waals interfaces, presumably leading to the different features for the A and B excitons, as discussed below.

**Figure 3:**
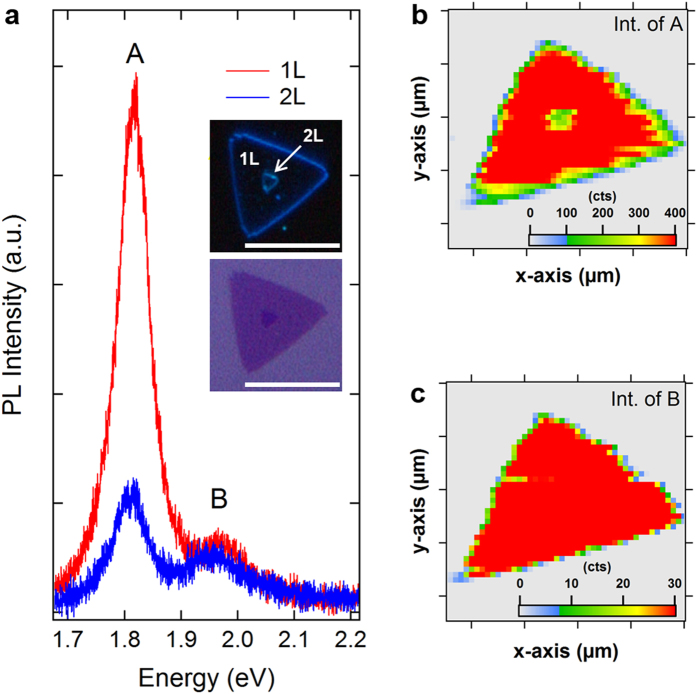
Photoluminescence intensity map of an individual CVD-MoS_2_ crystal. (**a**) Comparison of photoluminescence spectra measured at the 1L- and 2L-MoS_2_ regions of an individual CVD-MoS_2_ containing both 1L- and 2L-MoS_2_. The insets display the optical microscope images (upper: dark field, lower: bright field) of the measured CVD-MoS_2_ crystal, showing both the 1L- (large triangular base) and 2L-MoS_2_ (small triangular top in the center) regions. The scale bar in the inset images indicates 10 μm. (**b**,**c**) Photoluminescence intensity map showing the intensity distribution of the A exciton (**b**) and B exciton (**c**) for the individual CVD-MoS_2_ crystal.

To obtain further evidence for the distinct behavior of the A and B excitons with interlayer interactions, 2-dimensional mapping of photoluminescence intensity was carried out. Figure 3a shows the representative photoluminescence spectra taken from the 1L and 2L regions, in which an individual CVD-MoS_2_ contained both the 1L and 2L regions, as shown in the insets of Fig. 3a. A drastic change in the A exciton and the insensitivity of the B exciton with the interlayer interactions of the 2L-MoS_2_ crystals were consistently observed in the spectra. Figure 3b,c display the spatially resolved photoluminescence intensity map of the A and B excitons, respectively. The layer thickness of the CVD-MoS_2_ was confirmed by estimating the frequency difference between the A_1g_ and E^1^_2g_ phonon modes in the Raman spectra (see Supplementary Fig. S3). The intensity map of the A exciton correctly reproduces the 1L and 2L structures due to the large difference in the photoluminescence intensity from the 1L- and 2L-MoS_2_ (Fig. 3b). In contrast, the intensity map of the B exciton shows an almost uniform distribution over the whole MoS_2_ crystal, regardless of the layer number (Fig. 3c). Our observation clearly indicates that the emission quantum yield of the A exciton changes drastically with the interlayer interactions but that the influence on the B exciton is negligible, as seen in the above results of photoluminescence spectra.

**Figure 4:**
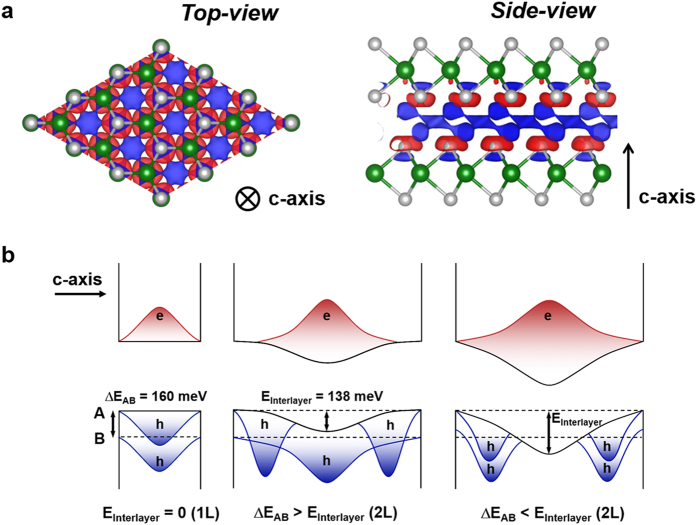
Calculated charge density difference and simplified band-bending model. (**a**) Top (left) and side (right) views of calculated charge density difference plots of 2L-MoS_2_. Red and blue regions depict the isosurface of electron accumulation and depletion at a value of 1.2 × 10^−4^ e/Å^3^, respectively. (**b**) Schematic energy band diagram of the 1L- (left panel) and 2L-MoS_2_ (middle and right panels) crystals, illustrating the quantum-confined Stark effect in 2L-MoS_2_ due to the interlayer interactions. E_Interlayer_ is the total energy difference due to the interlayer interactions in 2L-MoS_2_, and ΔE_AB_ is the energy difference between the A and B excitons. Distributions of electron (red) and hole (blue) wavefunctions are drawn within the quantum well.

To gain insight into the distinct behavior of the A and B excitons with interlayer interactions in the MoS_2_ crystals, we calculated the charge density difference (ρ_diff._) of the 2L-MoS_2_, as defined by ρ_diff._ = ρ_2L_ − ρ_1L-upper_ − ρ_1L-lower_, where ρ_2L_, ρ_1L-upper_, and ρ_1L-lower_ are the charge density of 2L-MoS_2_, isolated upper, and lower 1L-MoS_2_, respectively. Figure 4a displays the calculated charge density difference in the top-view (left) and side-view (right) in 2L-MoS_2_. The red and blue regions depict the isosurface of electron accumulation and depletion of valence electrons at a value of 1.2 × 10^−4^ e/Å^3^, respectively. As shown in Fig. 4a, a significant charge redistribution occurs near the van der Waals interface of the 2L-MoS_2_. Notably, the spatial separation of positive and negative charge regions is observed in the side-view (Fig. 4a), which gives rise to a local electric field near the interface. Under optical excitation to produce electron and hole pairs, electrons can accumulate at the interfacial surfaces, and holes can thus be pushed away from the interface due to the local electric field, leading to a spatial separation of electrons and holes in the out-of-plane direction. Here, we propose a simplified band-bending model to describe the spatial separation of electrons and holes due to the charge redistribution at the interface and, in turn, the distinct behavior of the A and B excitons with the interlayer interactions. Figure 4b presents a simplified band diagram in the out-of-plane direction, showing the band-bending of 2L-MoS_2_ depending on the degree of interlayer interactions, compared to that of 1L-MoS_2_ without the interlayer interactions. The local electric field results in a decrease in the overlap between electron and hole wavefunctions that triggers a reduced quantum yield of excitonic emission, which is analogous to the concept of the quantum-confined Stark effect in conventional quantum wells^19^.

Our proposed model describes a possible scenario to explain the distinct behavior of the A and B excitons with the interlayer interactions. When the band-bending energy is smaller than the difference between the A and B exciton energies, the hole wavefunction of only the A exciton is off-center of the quantum well, which leads to a drastic decrease in the emission intensity of the A exciton (middle panel of Fig. 4b). However, when the band-bending energy is larger, the hole wavefunctions of both the A and B excitons are forced toward the energy barriers, thereby decreasing the intensities of both the A and B excitons (right panel of Fig. 4b). To quantify the band-bending energy due to the interlayer interaction in 2L-MoS_2_, we estimated the total energy difference (E_Interlayer_) using DFT calculations, where E_Interlayer_ = E_2L_ − 2 × E_1L_, E_2L_ is the total energy of 2L-MoS_2_, and E_1L_ is the total energy of 1L-MoS_2_. As a result, we obtained an E_Interlayer_ of 138 meV, which is smaller than the energy difference between the A and B excitons, which is 160 meV. This indicates that the interlayer interactions in 2L-MoS_2_ can induce the band-bending by a smaller amount than the energy difference between the A and B excitons, which mostly affects the A exciton with the hole wavefunctions depleted at the center of the quantum well and results in a drastic decrease in the emission quantum yield with the interlayer interactions in 2L-MoS_2_. Note that the source of charge redistribution in 2L-MoS_2_ is purely van der Waals interaction in our calculations. Recent theoretical studies have shown that van der Waals interactions can induce sizable electronic charge redistribution and electrostatic moments in molecules and also in 2-dimensional layered phosphorus^20^,^21^. For example, the interlayer interaction energy in a bilayer boron nitride system was theoretically calculated to be as large as 100 meV^22^, which is very close to our value for 2L-MoS_2_. In our band-bending model, the calculated interlayer interaction energy of 138 meV is assigned to the band-bending energy because the interlayer interaction directly corresponds to the charge redistribution. In conventional semiconductor quantum wells, the quantum-confined Stark effect is accompanied by a red-shift of exciton energy due to the band-bending that is induced by the electric field^23^,^24^. For the 2L-MoS_2_, we also observed a red-shift in exciton energy by ~10 meV (see Supplementary Fig. S4). However, the red-shift in 2L-MoS_2_ can also be attributed to a decreased quantum confinement energy due to the larger width of the quantum well, whereas a reduced exciton binding energy in 2L-MoS_2_ can give rise to a blue-shift in exciton energy^25^,^26^. To quantitatively understand the shift in exciton energy with interlayer interactions, further study would be required with an elaborate experimental/theoretical approach. It is worthy to discuss the effect of SiO_2_ substrate. Recently, it has been experimentally observed that the photoluminescence intensity is significantly enhanced in suspended 1L-MoS_2_ compared to SiO_2_-supported monolayer MoS_2_, whereas the effect of substrate is relatively small in 2L-MoS_2_^18^. This suggests that van der Waals interaction even in 1L-MoS_2_ supported on SiO_2_ substrate can play a crucial role in introducing a charge redistribution and in changing the excitonic properties.

## Conclusion

In conclusion, we studied the distinct behavior of the A and B excitons in the presence of interlayer interactions by examining the excitonic properties of the 1L- and 2L-MoS_2_ crystals. A spatially resolved micro-photoluminescence map of the A and B excitons revealed that the emission quantum yield of the A exciton is drastically decreased with the interlayer interactions in 2L-MoS_2_, whereas that of the B exciton is nearly constant for both 1L- and 2L-MoS_2_. By calculating the charge density difference in 2L-MoS_2_, we observed a significant charge redistribution due to the interlayer interactions in 2L-MoS_2_, giving rise to a local electric field at the interfacial region. In an attempt to explain the distinct behavior of the A and B excitons, we proposed a band-bending model that is analogous to the quantum-confined Stark effect in conventional quantum wells. Our proposed model suggests that the A exciton is mostly influenced by the interlayer interaction because the band-bending energy (138 meV) from the local electric field is smaller than the energy difference between the A and B excitons (160 meV). Our study implies that the interlayer interactions in the van der Waals structures can play a critical role in tuning the excitonic properties and should be carefully considered for designing future optoelectronic devices based on the homo- and hetero-structures of two-dimensional materials.

## Methods

### Preparation of atomic-layered MoS_2_ crystals

Atomic-layered MoS_2_ crystals were prepared by both mechanical exfoliation (ME) and chemical vapor deposition (CVD) techniques. ME-MoS_2_ was mechanically exfoliated from natural bulk crystal (MoS_2_ Crystals), and transferred onto a Si wafer with a 400 nm-thick SiO_2_ top layer that was fabricated by dry thermal oxidation. The CVD-MoS_2_ was synthesized on a 270-nm-thick SiO_2_/Si substrate using the chemical vapor deposition technique (commercially available from HQ Graphene).

### Optical measurement

Micro-photoluminescence and Raman spectroscopic measurements were carried out at room temperature using a home-built optical microscope equipped with a 20×, 0.45NA objective (Nikon). Spatial resolution of the optical micro-spectroscopy was estimated to be approximately 400 nm. A continuous wave argon-ion laser (Coherent), which was tuned at a wavelength of 457.9 nm, was focused to a beam spot size of ~1 μm to excite the MoS_2_ crystals on the sample. A relatively low laser power of 60 μW (on the sample surface) was used to avoid degrading the samples with heating. Photoluminescence spectra were collected using a spectrometer (Acton) and a cooled CCD (charge-coupled device) (PIXIS:2KB excelon, Princeton Instruments) with a spectral resolution of 0.1 nm.

### Theoretical calculation

The electronic band structure calculations that considered the spin-orbit interactions were performed using the projector augmented wave (PAW) method^27^,^28^ as implemented in the Vienna ab initio simulation package (VASP)^29^. Plane waves with an energy cut-off of 500 eV were used for the expansion of the Kohn-Sham orbitals. A vacuum space of more than 15 Å was employed in all calculations, along with a 24 × 24 × 1 Monkhorst-Pack grid mesh for integrals in the 2D Brillouin zone. For exchange-correlation interactions, the functional of Perdew *et al*.^30^, a form of the generalized gradient approximation (GGA), was used. For both 1L- and 2L-MoS_2_, experimentally measured lattice constants of the bulk MoS_2_ (*a* = 3.16 Å) in the lateral direction were adopted and all of the atomic positions were optimized using the conjugate gradient method. Furthermore, for 2L-MoS_2_, the van der Waals (vdW) interactions were considered^31^.

## Additional Information

**How to cite this article**: Kim, J. G. *et al*. Effect of interlayer interactions on exciton luminescence in atomic-layered MoS_2_ crystals. *Sci. Rep.* **6**, 29813; doi: 10.1038/srep29813 (2016).

## Supplementary Material

Supplementary Information
