# Supplementary material for: Effect of interlayer interactions on exciton luminescence in atomic-layered MoS2 crystals
Source: Sci Rep. 2016 Jul 15;6:29813. doi: 10.1038/srep29813 (PMC4945952; doi:10.1038/srep29813)
Supplement: Supplementary Information [file srep29813-s1.pdf]

Supplementary Information for

Effect of interlayer interactions on exciton luminescence in atomic-layered MoS_2 crystals

Jung Gon Kim, Won Seok Yun, Sunghwan Jo, JaeDong Lee* and Chang-Hee Cho*

Department of Emerging Materials Science, DGIST, Daegu 42988, South Korea

*Corresponding authors. E-mail: jdlee@dgist.ac.kr, chcho@dgist.ac.kr

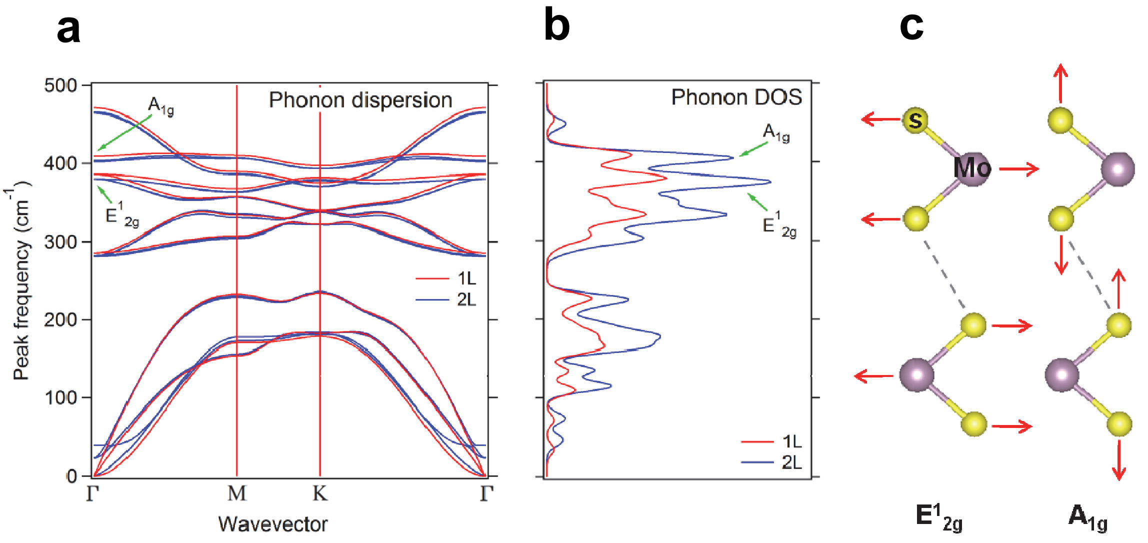

Figure S1. a and b, Calculated phonon dispersion **(a)** and density of states **(b)** of 1L- and 2L-MoS_2 crystals [1]. **c**, Atomic displacements corresponding to E^1_{2g} (in-plane) and A_{1g} (out-of-plane) modes.

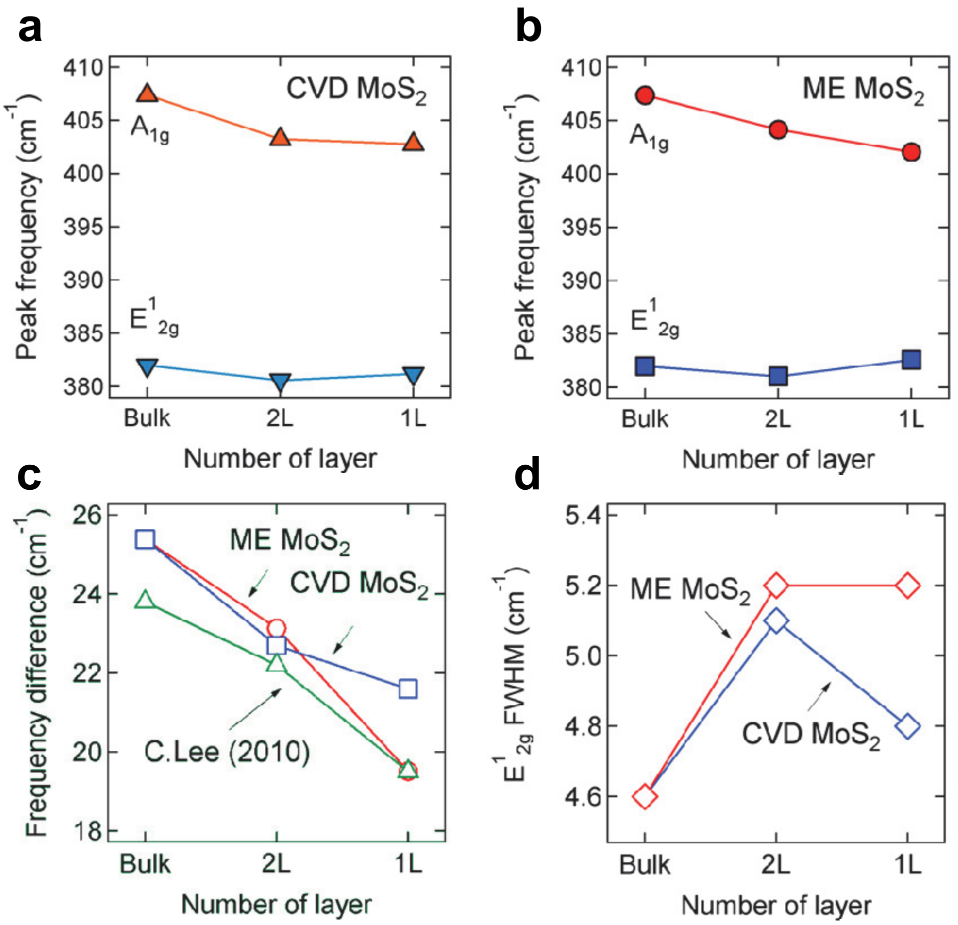

Figure S2. a and b, Plot of Raman peak frequency of CVD-MoS_2 **(a)** and ME-MoS_2 **(b)** as a function of number of layers. **c**, Frequency difference between A_{1g} and E^1_{2g} modes as a function of number of layers. The frequency difference taken from ref. [2] is also plotted for a comparison. **d**, Spectral line width (FWHM) of E^1_{2g} mode as a function of number of layers.

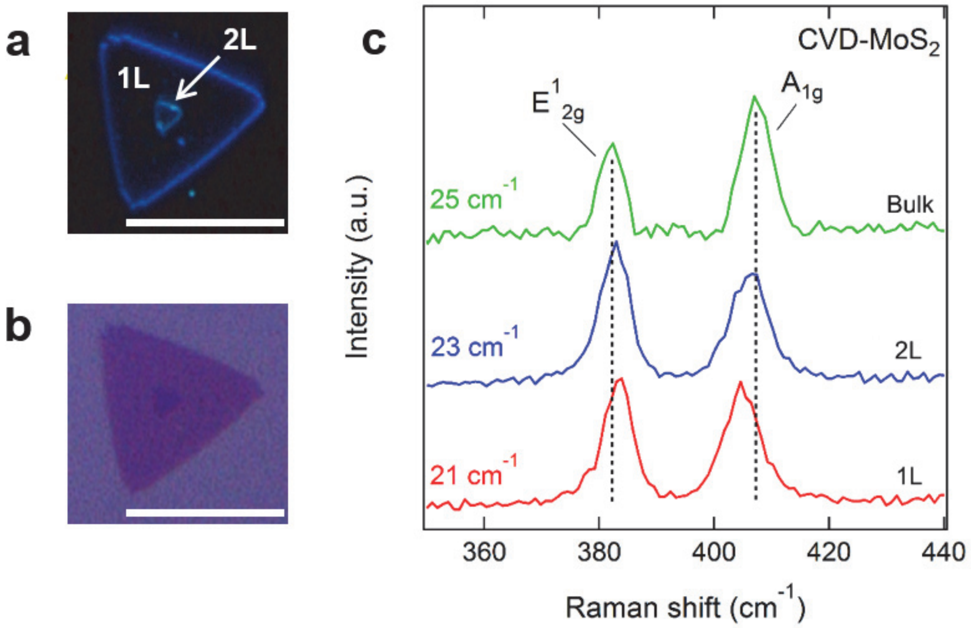

Figure S3. Comparison of Raman spectra between 2L- and 1L-MoS_2 grown by CVD method. **a and b**, Dark **(a)** and bright **(b)** field optical microscope images of CVD-MoS_2, which contains both 1L (large triangular base) and 2L (small triangular top at the center) regions. Scale bar indicates 10 μm. **c**, Raman spectra measured on the 2L and 1L regions of CVD-MoS_2 crystal. Raman spectra of bulk MoS_2 is also plotted for a comparison. The estimated frequency difference between A_{1g} and E^1_{2g} modes is denoted on the spectra.

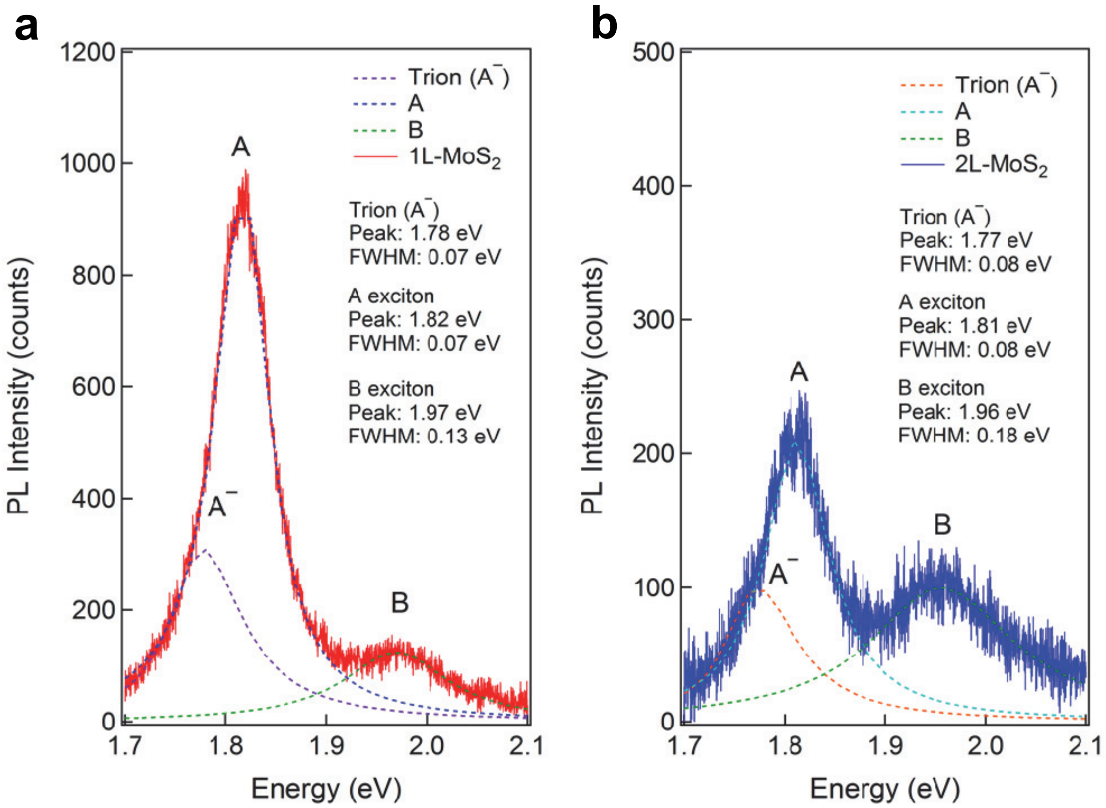

Figure S4. a and b, Photoluminescence spectra of 1L- **(a)** and 2L-MoS_2 **(b)** crystals, showing the trion (A^-), A exciton, and B exciton peaks. Dashed lines are Lorentzian fits to the measured spectra.

References

[1] Togo, A. *et al.* First-principles calculations of the ferroelastic transition between rutile-type and $CaCl_2$-type SiO_2 at high pressures. *Phys. Rev. B* **78**, 134106 (2008).

[2] Lee, C. *et al*. Anomalous lattice vibrations of single- and few-layer MoS_2. *ACS Nano* **4**, 2695 - 2700 (2010).
